# Transcriptomic data of *Salmonella enterica* subsp. *enterica* serovar Typhimurium str. 14028S treated with novobiocin

**DOI:** 10.1016/j.dib.2020.105297

**Published:** 2020-02-17

**Authors:** Natalia E. Gogoleva, Tatiana A. Konnova, Alexander S. Balkin, Andrey O. Plotnikov, Yuri V. Gogolev

**Affiliations:** a Kazan Institute of Biochemistry and Biophysics, Kazan Scientific Center of RAS, 2/31 Lobachevsky St., Kazan 420111, Russian Federation; b Institute of Fundamental Medicine and Biology, Kazan (Volga Region) Federal University, 18 Lenina St., Kazan 420021, Russian Federation; c Center of Shared Scientific Equipment “Persistence of Microorganisms”, Institute for Cellular and Intracellular Symbiosis, Ural Branch of Russian Academy of Sciences, Orenburg, Russian Federation

**Keywords:** *Salmonella enterica*, Novobiocin treatment, RNA-seq, Illumina

## Abstract

In enteric bacteria, DNA supercoiling is highly responsive to environmental conditions. Host specific features of environment serve as cues for the expression of genes required for colonization of host niches via changing supercoiling [1]. It has been shown that substitution at position 87 of GyrA of *Salmonella enterica* str. SL1344 influences global supercoiling and results in an altered transcriptome with increased expression of stress response pathways [2]. Aminocoumarin antibiotics, such as novobiocin, can be used to relax supercoiling and alter the expression of supercoiling-sensitive genes. Meanwhile, *Salmonella enterica* demonstrates a significant resistance to this antibiotic and relatively small variability of supercoiling in response to the growth phase, osmotic pressure, and novobiocin treatment. Here we present for the first time transcriptome data of *Salmonella enterica* subsp*. Enterica* serovar Typhimurium str. 14028S grown in the presence of novobiocin. These data will help identify genes involved in novobiocin resistance and adaptation processes associated with torsion perturbations in *S*. *enterica*. Cleaned FASTQ files for the RNA-seq libraries are deposited in the NCBI Sequence Read Archive (SRA, Identifier: SRP239815) and have been assigned BioProject accession PRJNA599397.

**Table:** Specifications Table

Subject	Biochemistry, Genetics and Molecular Biology: Molecular Biology
Specific subject area	Transcriptomics
Type of data	Transcriptome sequences, table, figure
How data were acquired	High-throughput RNA-sequencing
Data format	Raw reads filtered and analysed with statistical tests, FASTQ
Parameters for data collection	Comparison of *S*. *enterica* control and treated samples following treatment with novobiocin
Description of data collection	RNA from control and novobiocin treated samples subjected to RNA-Sequencing and transcriptome profiling
Data source location	Kazan Scientific Centre of RAS, Kazan, Russia.
Data accessibility	Cleaned FASTQ files are deposited in a public repository: Repository name: NCBI Sequence Read Archive (SRA) Data identification number: PRJNA599397 Direct URL to data: https://www.ncbi.nlm.nih.gov/bioproject/PRJNA599397/

**Table:** 

**Value of the Data** • Resistance of bacterial pathogens to current antibiotics is an increasingly urgent worldwide problem. • Host specific environmental conditions cause increased DNA supercoiling in enteric bacteria [1]. Many promoters of genes required for colonization of host niches respond to changes in the supercoiling state and apparently are sensitive to the novobiocin treatment [2,3]. • The transcriptome data set generated for control and novobiocin treated samples of *S. enterica* str. 14028S helps in identification of differentially expressed genes for a better understanding of the molecular mechanism of the bacterial signaling and antibiotic resistance.

## 1 Data description

**Fig. 1:**
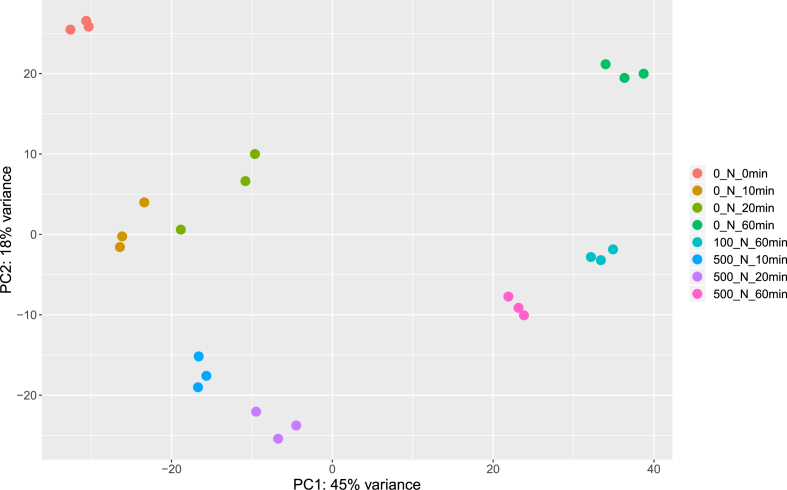
Principal component analysis (PCA) of the general transcriptome characteristics. The first principal component (component 1) accounted for 45% and the second principal component (component 2) for 18% of the total variance in the dataset. Legend description: “0_N_0min”, “0_N_10min”, “0_N_20min” and “0_N_60min” – samples of cultures non treated with novobiocin and grown 0, 10, 20, 60 min respectively; “500_N_10min”, “500_N_20min” and “500_N_60min” – samples of cultures treated with novobiocin at a concentration of 500 μg per mL, and incubated 10, 20, 60 min respectively; “100_N_60min” – samples of cultures treated with novobiocin at a concentration of 100 μg per mL, followed by incubation for 60 minutes.

The dataset of this article provides information on raw RNA-seq reads obtained from samples of *S. enterica* cultures treated with novobiocin at concentrations of 100 and 500 μg per mL, and untreated cultures. The data sets were named based on novobiocin concentration and growth time after addition of novobiocin. Information on the treatment (sampling) time and growth rate of the cultures is presented in Table 1. This table also provides the NCBI SRA accession numbers of the cleaned FASTQ files for all biological replicates. Coverage estimates and reads mapping statistics are summarized in Table 2. PCA plot of RNA-seq data presented in Fig. 1 demonstrates the variance between sample groups and sample replicates according to gene expression levels.

**Table 1:** Samples of the *Salmonella enterica* subsp. *enterica* serovar Typhimurium str. 14028S cultures treated with novobiocin.

Biological replicates	Novobiocin concentration, μg/mL	Duration of cultivation, min	Culture density, OD	NCBI SRA accession number
0_N_0min_1	0	0	0.60	SRX7514156
0_N_0min_2			0.58	SRX7514157
0_N_0min_3			0.57	SRX7514158
0_N_10min_1		10	0.82	SRX7514172
0_N_10min_2			0.80	SRX7514173
0_N_10min_3			0.78	SRX7514174
0_N_20min_1		20	1.41	SRX7514175
0_N_20min_2			1.43	SRX7514154
0_N_20min_3			1.38	SRX7514155
0_N_60min_1		60	2.22	SRX7514159
0_N_60min_2			1.73	SRX7514160
0_N_60min_3			2.39	SRX7514161
100_N_60min_1	100u	60	1.48	SRX7514162
100_N_60min_2			1.55	SRX7514163
100_N_60min_3			1.53	SRX7514165
500_N_10min_1	500	10	0.75	SRX7514152
500_N_10min_2			0.69	SRX7514153
500_N_10min_3			0.69	SRX7514164
500_N_20min_1		20	1.21	SRX7514169
500_N_20min_2			1.21	SRX7514170
500_N_20min_3			1.17	SRX7514171
500_N_60min_1		60	1.32	SRX7514166
500_N_60min_2			1.37	SRX7514167
500_N_60min_3			1.25	SRX7514168

**Table 2:** Cleaned reads and reads mapped on reference genome.

Library	Number of cleaned reads	Number of reads mapped on genome	% Mapped reads
0_N_0min_1	10,130,456	10,090,677	99.60
0_N_0min_2	12,053,713	12,017,895	99.70
0_N_0min_3	9,335,698	9,305,851	99.68
0_N_10min_1	3,852,431	3,611,345	93.74
0_N_10min_2	4,481,893	4,216,603	94.08
0_N_10min_3	8,060,919	7,915,760	98.20
0_N_20min_1	2,886,140	2,704,610	93.71
0_N_20min_2	10,097,850	10,043,330	99.46
0_N_20min_3	8,459,432	8,320,338	98.36
0_N_60min_1	13,430,409	13,384,219	99.65
0_N_60min_2	12,589,998	12,528,485	99.51
0_N_60min_3	10,850,083	10,812,960	99.66
100_N_60min_1	10,189,937	10,160,243	99.71
100_N_60min_2	10,194,538	10,154,167	99.60
100_N_60min_3	12,298,443	12,208,069	99.27
500_N_10min_1	2,841,461	2,641,102	92.95
500_N_10min_2	761,955	564,342	74.07
500_N_10min_3	2,011,352	1,741,299	86.57
500_N_20min_1	2,457,816	2,197,352	89.40
500_N_20min_2	1,668,653	1,453,689	87.12
500_N_20min_3	2,760,241	2,559,427	92.72
500_N_60min_1	9,993,333	9,952,597	99.59
500_N_60min_2	9,569,160	9,535,373	99.65
500_N_60min_3	10,908,863	10,846,616	99.43

## 2 Experimental design, materials, and methods

### 2.1 Bacterial strains and growth conditions

Strain used in this work was *Salmonella enterica* subsp. *enterica* serovar Typhimurium ATCC 14028S (NCBI:txid588858). This strain was cultured on a Luria-Bertani (LB) agar plate (Sigma Aldrich) and a single colony was used to inoculate 10 mL of fresh LB broth for aerobic incubation at 37 °C. After 12 h, 1 mL of this preculture was added to 50 mL of LB broth and incubated aerobically at 37 °C until the middle of the exponential growth phase (A600 = 0.6), which was reached approximately 4 h after the initial inoculation. Novobiocin (Sigma Aldrich) was added at various concentrations directly to the flask and turbidity was monitored by measuring the optical density.

### 2.2 Experiment design

The bacteria were grown in LB medium until middle log-phase where 0, 100 and 500 μg of novobiocin per mL was added. This increase in the antibiotic concentration has led to a gradual inhibition of the *Salmonella* growth, but by the end of time sampling the growth of bacteria in both cases resumed. At time zero and after 60 min 10 mL aliquots were taken and bacteria were harvested and fixed. The control sample and the sample supplemented with 500 μg of novobiocin per mL were also fixed after 10 and 20 minutes. Total RNA was isolated and cDNA libraries were prepared for RNA-sequencing. Directional libraries were sequenced on Illumina Hiseq2500 in single reads. The RNA-seq raw reads were further analyzed to get the clean reads and stored in FASTQ files.

### 2.3 Library construction and sequencing

Bacterial cultures were fixed with an equal volume of cold RNA-stabilizing solution (19% ethanol, 1% acidic phenol, pH 5.5) on ice for 30 minutes. Cells were harvested by centrifugation and total RNA was extracted using RNA Extract Reagent (Evrogen, Russia) according to the manufacturer's protocol. DNA contaminants were removed using RNase-free DNase I kit (Ambion, USA). The integrity of the RNA was checked by Agilent 2100 bioanalyzer (USA). rRNA depletion performed using Ribo-Zero rRNA Removal Kit for Gram-Negative Bacteria (Illumina, USA). Barcoded RNA libraries were generated. NEBNext Ultra Directional RNA Library Prep Kit for Illumina was used to prepare RNA-seq libraries. The resulting average size of the cDNA libraries was approximately 300 bp. Libraries were sequenced using the Illumina HiSeq 2500 sequencing platform.

### 2.4 Sequence QC and filtering

266,853,687 reads were obtained in total with a length of 60 nucleotides. Raw Fastq files and clean reads were quality controlled using FastQC software (Version 0.11.5) [4]. Raw reads were filtered using BBDuk (v. 37.23, http://jgi.doe.gov/data-and-tools/bb-tools/) to remove Illumina adapters, NEB indexes and to eliminate rRNA reads (ktrim = r k = 23 mink = 11 hdist = 1 tpe tbo minlen = 25).

### 2.5 Reads alignment to the reference genome

Filtered high-quality reads were mapped onto the genome sequence of the *Salmonella enterica* subsp. *enterica* serovar Typhimurium strain 14028S assembly GCA_000022165.1 (ftp://ftp.ncbi.nlm.nih.gov/genomes/all/GCF/000/022/165/GCF_000022165.1_ASM2216v1/GCF_000022165.1_ASM2216v1_genomic.fna.gz).

HISAT2 version 2.1.0 [5] was used to build index of reference genome and align clean reads to genome with the following parameters: hisat2 -p --dta -x -U –S. SAM files of alignments created by HISAT2 were converted to BAM files using SAM-tools view [6]. Coverage estimates and reads mapping statistics are presented in Table 2. Mapped reads were summarized at the transcript level into a count matrix using the assembler of RNA-Seq alignments StringTie [7]. DESeq2 [8] was used to assess variance between sample groups and sample replicates using principal component analysis (PCA). PCA plot shown in the Fig. 1 demonstrates the overall quality of our sample collection, library preparation, and sequencing.
